# Colorenal fistula after renal tumour cryotherapy

**DOI:** 10.1016/j.ijscr.2018.11.050

**Published:** 2018-11-24

**Authors:** Maialen Mozo, Rubén Gonzálo, Jose Manuel Gutiérrez, Luis Eloy Gutiérrez, Luiza Cotruta, Antonio Roca, Roberto García

**Affiliations:** a Department of General Surgery of Sierrallana Hospital, Torrelavega, Cantabria, Spain; b Department of Urology of Mompia Clinic, Santa Cruz de Bezana, Cantabria, Spain; c Department of Radiology of Mompia Clinic, Santa Cruz de Bezana, Cantabria, Spain

**Keywords:** Colorenal fistula, Cryoablation, Renal tumour, Urinary tract infection, Pneumaturia, Fistulectomy

## Abstract

**Introduction:**

Computed tomography (CT)-guided percutaneous cryoablation is increasingly utilized for renal cell carcinoma. Bowel injury is a known complication but is extremely rare. We herein present the case of a 58-year-old man diagnosed with a colorenal fistula after cryoablation of a left renal tumour.

**Presentation of Case:**

A left renal tumour was incidentally found on abdominal CT examination performed for a slight increase in transaminases. Abdominal ultrasonography revealed a 31 × 32-mm solid, well-defined, cortical tumour at the lower pole of his left kidney. The patient was asymptomatic and had no distant metastasis. The decision was made to treat the tumour with percutaneous cryoablation, with good response to the technique. Two months later, the patient had recurrent urinary tract infections and pneumaturia. In the absence of improvement with antibiotic treatment, CT was performed and revealed a fistula connecting the descending colon and renal parenchyma. The decision was made to perform surgery to repair the defect caused by percutaneous cryotherapy.

**Discussion:**

To reduce adverse effects of the procedure and preserve renal function, percutaneous ablation techniques have been developed. Internal injury is a known complication and it is particularly common in cases of renal tumours located in the upper and anterior kidney. The diagnosis is based on symptoms and imaging. Most colorenal fistulas have been treated conservatively with good results.

**Conclusion:**

The patient recovered from surgery and was discharged with no complications.

## Highlights

• Percutaneous ablation techniques have been developed to treat renal tumours.
• Bowel lesions after cryoablation are diagnosed using symptoms and imaging.
• Most colorenal fistulas are treated conservatively with good results.
• Surgical treatment is reserved for complicated or persistent colorenal fistulas.

## 1 Introduction

This work has been reported in line with the SCARE criteria [1].

Percutaneous ablation techniques are now used to treat some solid visceral neoplasms [2]. These techniques include radiofrequency, cryoablation, and microwave ablation. A variety of different imaging modalities have been used for guidance for ablation, including ultrasound (both percutaneous and intraoperative), CT, CT fluoroscopy, magnetic resonance imaging (MRI), and, occasionally, plain films/fluoroscopy. The modality used for guidance is heavily influenced by both the location of the tumour and the local availability of equipment. For small renal tumours, cryoablation offers promising results with a low complication rate [3,4]. Complications are a potential risk with any invasive procedure and although bowel injury is a known complication of the technique, it is extremely rare [5,6].

## 2 Presentation of case

**Fig. 1:**
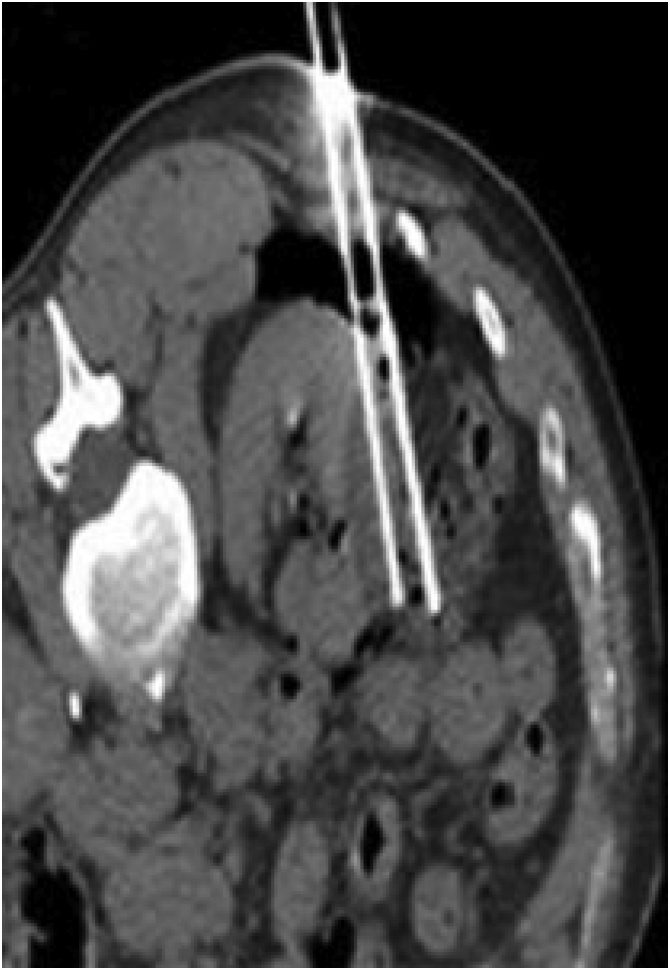
Percutaneous cryoablation monitoring of the cortical tumour at the lower pole of the left kidney using computed tomography.

**Fig. 2:**
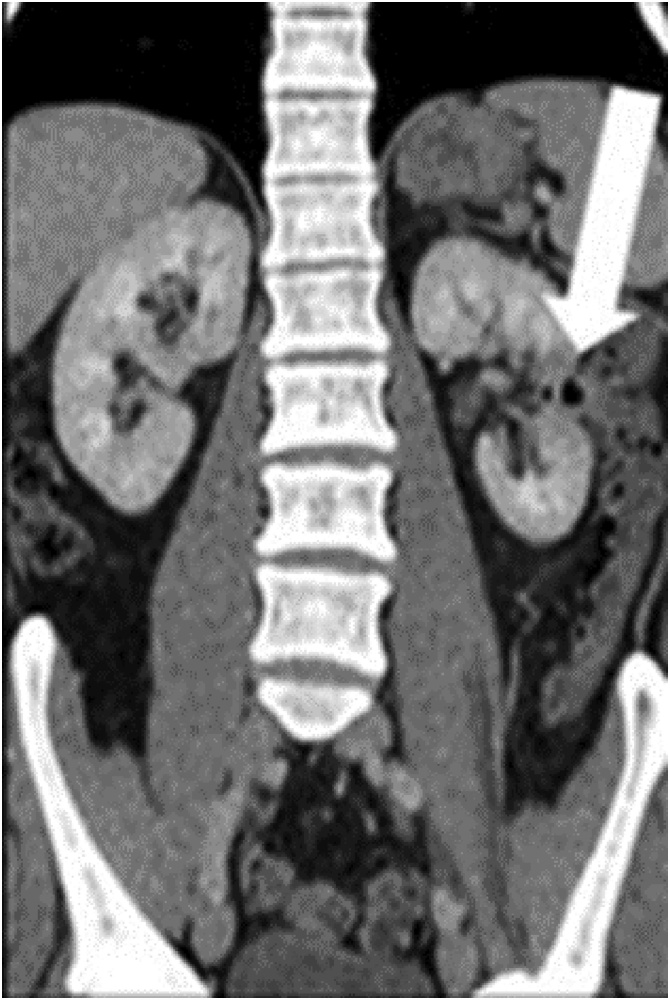
Abdominal CT with intravenous contrast. Left colorenal fistula after renal tumour cryotherapy.

**Fig. 3:**
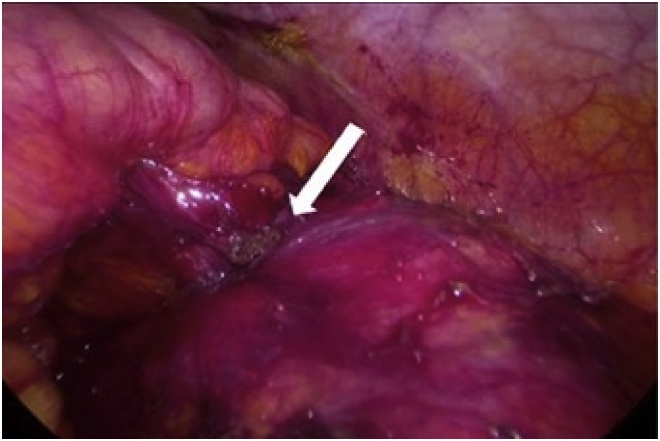
Left colorenal fistula viewed on laparoscopy.

A 58-year-old man with no significant history was diagnosed with left renal carcinoma. Although asymptomatic, he underwent abdominal ultrasonography due to a slight increase in transaminases, which revealed a 31 × 32-mm solid, well-defined, cortical tumour at the lower pole of his left kidney. Thoracoabdominal computed tomography (CT) confirmed the presence of a 35-mm, well-defined, heterogeneous, anterior mesorenal mass with increased uptake and malignant appearance in the left kidney, but no extracapsular extension, fat infiltration, or perirenal adenopathy. The study was completed with urinary cytology, which proved negative for urothelial tumour cells. The decision was made to treat the tumour with percutaneous cryoablation (Fig. 1). During the cryoablation technique, there was suspicion of a possible intestinal perforation seen on CT. Therefore, abdominal ultrasonography was performed, but no changes were identified. Two months later, he developed recurrent urinary tract infections, with pneumaturia and urine culture positive for *Escherichia coli*, which did not improve despite antibiotic therapy. An abdominal CT with intravenous contrast was performed, which revealed a segment of descending colon in contact with the anterior face of the left kidney, with air bubbles in the renal parenchyma, left renal calyces, and bladder (Fig. 2). He was diagnosed with a colorenal fistula after cryoablation. The possibility of performing a CT urography was considered to complete the study, but the patient rejected the test. In view of the failure of medical treatment, surgery was performed with laparoscopy. The colorenal fistula was located in the descending colon, as revealed on CT (Fig. 3). The colon was released from the renal parenchyma with scissors and monopolar coagulation. Both the renal and colonic orifices were sutured with absorbable monofilament stitches and omental transposition was performed. Intestinal resection was not necessary because of the good condition of the colon and a double J stent was not placed. The patient was discharged with no complications. He remains asymptomatic and has negative urine cultures.

## 3 Discussion

The incidence of renal cell carcinoma has been increasing in recent years. The preferred treatment has been radical or partial nephrectomy. To reduce adverse effects of the procedure and preserve renal function, percutaneous ablation techniques have been developed for the treatment of these tumours with excellent results [2]. The primary techniques are cryoablation, radiofrequency ablation, percutaneous ethanol injection, and microwave ablation. Improvement in technique has led to an increase in their use for small renal tumours. Cryoablation has shown that low temperatures can be applied for tissue destruction and can be used with an open, laparoscopic, or percutaneous approach. During cryoablation, a cryogenic probe is inserted in the target tissue, and liquid gas (argon) is used to cool it rapidly, forming an ice ball around the probe that thickens as the procedure progresses (cell death depends on the time and temperature) [7]. The low temperatures reached in the kidney can be transferred to adjacent organs such as the colon, duodenum, or ureter, which leads to serious complications. Owing to the proximity of the cryoablation site to the kidney, intestinal injury is a known complication, particularly in cases of tumours located in the upper and anterior kidney. Intestinal injury is a rare complication, accounting for only 0–1% of injuries at this level [8]. If not enough fat is present between the tumour and the intestine (minimum of 5 mm), different manoeuvres have been described to separate the kidney from the colon, including postural displacement, hydrodissection with 5% dextrose, injection of carbon dioxide, or balloon interposition [9].

Imaging methods are used to determine the extent to which freezing is appropriate. Better monitoring of the procedure is obtained with CT or magnetic resonance imaging (MRI) than with ultrasonography. CT allows for easy, fast, and accurate visualization of the ablation area by decreasing the attenuation of frozen tissue. MRI allows for manual displacement of the lesion without the operator being exposed to radiation. However, artefacts will appear in the image because of air, movement, and the metal cryoprobe. Although the advantages of this technique are greater than those of CT, it is not available at our center [7].

The diagnosis of bowel lesions after cryoablation is based on symptoms and imaging. There may be an interval of days or weeks between cryoablation and the onset of symptoms. Urinary symptoms and pneumaturia were the characteristic symptoms that made us suspect the possibility of a colorenal fistula in our patient. Although the follow-up ultrasonography after the procedure showed no immediate complications, the symptoms and abdominal CT proved diagnostic.

No data are available to identify optimal treatment. Different options include both conservative treatment (antibiotic therapy, percutaneous abscess drainage, and therapeutic colonoscopy) and surgical treatment (laparoscopy or laparotomy with or without nephrectomy and/or colectomy). Most colon lesions have been treated conservatively with good results [6,10]. Emergency surgery should be considered when damage to the colon causes obstruction, perforation, or severe sepsis [6]. In our case, antibiotic treatment was tried, but owing to its failure, the decision was made to perform surgery. Given the good condition of the patient, fistulectomy and epiploic repair were performed, reserving the most aggressive techniques for use in the event of failure or complication. The patient progressed without complications and was discharged. He is currently asymptomatic.

## 4 Conclusion

Cryoablation of renal tumours is a safe, low-risk technique with few complications. We should suspect a colorenal fistula in the presence of repeated urinary tract infections and pneumaturia in patients undergoing renal cryoablation. The treatment is controversial. If possible, conservative medical treatment should be used, reserving surgery for complicated or persistent colorenal fistulas.

## Conflict of interest

All authors declare no conflicts of interest associated with this manuscript.

## Funding sources

This research did not receive any specific grant from funding agencies in the public, commercial, or not-for-profit sectors.

## Ethics approval

The study is exempt from ethics approval by our institution, as the case was managed as per standard guidelines and no modification or experimental intervention was employed.

## Consent

Written informed consent was obtained from the patient for publication of this case report and accompanying images.

## Author contribution

M. M. acquired the data and wrote the article. R.G., J.M.G., L.E.G., L.C., A.R. and

R.G. coordinated and critically revised the study. All read and approved the final manuscript.

## Registration of research studies

There is no registration.

## Guarantor

M.M.

## Provenance and peer review

Not commissioned, externally peer reviewed.
